# Pulpotomy for treating primary teeth with irreversible pulpitis: a call for action

**DOI:** 10.1007/s40368-026-01199-7

**Published:** 2026-04-11

**Authors:** Nebu Philip, Mandeep Duggal, Hani Nazzal

**Affiliations:** 1 College of Dental Medicine, QU-Health, Qatar University, Doha, Qatar; 2 King’s College London, London, UK; 3 Hamad Dental Centre, Department of Paediatric Dentistry, Hamad Medical Corporation, Doha, Qatar; 4 College of Dental Medicine, QU-Health, Qatar University, Doha, Qatar

**Keywords:** Irreversible pulpitis, Pulpotomy, Primary teeth, Vital pulp therapy

## Abstract

**Purpose:**

This narrative review aims to assimilate the current state of evidence for considering pulpotomy as a treatment option to manage vital primary teeth clinically diagnosed with irreversible pulpitis.

**Methods:**

A structured literature search was undertaken on four electronic databases (MEDLINE, Embase, Web of Science, and Cochrane Library) to compile evidence for this narrative review. Eligible studies included clinical investigations and systematic reviews assessing outcomes of pulpotomy for treating primary teeth with clinical signs and symptoms of irreversible pulpitis.

**Results:**

Seven clinical studies and two meta-analyses met the inclusion criteria for data synthesis. Across the clinical studies,clinical success of pulpotomy for management of irreversible pulpitis-affected primary teeth ranged from 95-100%, while radiographic success ranged from 90-100% at the 6-month to 1-year post-treatment follow-up. The meta-analyses of these clinical studies concluded that pulpotomy may represent a viable alternative to conventional pulpectomy for the management of irreversible pulpitis in primary teeth. Based on current evidence, new diagnostic considerations and treatment procedural guidelines have been proposed for primary tooth pulpitis.

**Conclusions:**

Accumulating histological and clinical evidence supports pulpotomy as a successful, minimally invasive treatment option for management of primary teeth with clinical symptoms of irreversible pulpitis. The paper calls for revision of international guidelines to incorporate these developments and reduce unnecessary invasive interventions.

**Supplementary Information:**

The online version contains supplementary material available at 10.1007/s40368-026-01199-7.

## Introduction

Long-established treatment doctrines and practices need to change with the evolution of evidence-based knowledge. In recent years, a growing body of clinical and histological evidence has challenged traditional dogmas pertaining to pulpotomy and irreversible pulpitis. The Glossary of Endodontic Terms includes pulpotomy under vital pulp therapy (VPT) procedures that aims at “preserving and maintaining pulp tissue that has been compromised by trauma, caries, or restorative procedures in a healthy state” (AAE 2020). This definition reinforces the long-held notion that pulpotomy on teeth with deep caries lesions should be restricted to vital teeth which are asymptomatic or show only symptoms of reversible pulpitis (Boyer and Nguyen 2023). A diagnosis of irreversible pulpitis implies a state of vital pulp undergoing degenerative inflammation incapable of healing, and if left untreated, would result in pulpal necrosis. Accordingly, for several decades, the definitive treatment for carious primary or permanent teeth clinically diagnosed with irreversible pulpitis was either pulpectomy or extraction. This understanding is reflected in paediatric dentistry practice guidelines that recommend pulpectomy for conserving primary teeth diagnosed with irreversible pulpitis (Rodd et al. 2006; Duggal et al. 2022; Academy and of Pediatric Dentistry (AAPD), 2025; ; ).

The past decade has seen a paradigm shift towards conservative treatment approaches for the management of permanent teeth clinically diagnosed with irreversible pulpitis. The European Society of Endodontology (ESE) (Duncan et al. 2019, 2023) and the American Association of Endodontists (AAE 2021) have released position statements and S3-level clinical practice guidelines suggesting that “pre-treatment diagnosis of irreversible pulpitis is not necessarily an indication for pulpectomy”. A similar change is currently underway for the management of primary teeth diagnosed with irreversible pulpitis, with pulpotomy offering several advantages over the conventional pulpectomy or extraction treatment options. For instance, the ribbon-like torturous root canal anatomy encountered in primary molars often complicates radicular pulp extirpation and canal preparation during the pulpectomy procedure; other complications include possible extrusion of root filling material beyond the primary root apex and the relatively longer procedural time required (Coll et al. 2020). These pulpectomy-associated procedural difficulties can be avoided if pulpotomy is the treatment of choice. Extraction of pulpitis-affected primary molars is not preferred as it can result in loss of function and arch space integrity (Splieth et al. 2025). There is now evidence demonstrating that pulp therapy offers superior patient-centred outcomes for treating primary teeth compared to extraction (Abanto et al. 2024; Vitali et al. 2025).

The aim of this narrative review was to assimilate the current state of histological and clinical evidence for considering pulpotomy as a treatment option to manage primary teeth clinically diagnosed with irreversible pulpitis. The paper further proposes new diagnostic considerations specific for primary tooth pulpitis and suggests evidence-based procedural guidelines to be followed when considering pulpotomy for treating primary teeth clinically diagnosed with irreversible pulpitis.

## Primary pulp histology and defence mechanisms

Histological assessment has traditionally been the gold standard for appraisal of pulpal health (Seltzer et al. 1963; Dummer et al. 1980). Historically, the permanent dental pulp was believed to be very vulnerable to tissue insult from bacterial carious attack and the resulting inflammation. The lack of collateral circulation was thought to limit the ability of the pulp tissue to accommodate increases in intra-pulpal pressure or effectively deliver humoral and cellular immune components to the injured site (Tønder 1983). However, there is clear evidence that the permanent dental pulp can not only accommodate moderate increases in intra-pulpal pressure (Van Hassel 1971; Heyeraas and Berggreen 1999), but also has an effective immune defence response (Jontell et al. 1998; Farges et al. 2015; Lundy et al. 2020), and an innate ability to heal itself if the tissue insult is removed (Mjör and Tronstad 1974). Histopathological and histobacteriological studies in permanent teeth have shown healthy pulp architecture, free from inflammation and bacteria, a few millimetres away from the bacterially colonised ‘irreversibly’ inflamed pulp tissue (Ricucci et al. 2019; Demant et al. 2021). These histological studies indicate that irreversible pulpitis is a spatially and temporally graded disease with the bacterially contaminated pulp eliciting a severe local inflammatory response, but tissues apical to these inflamed areas appear histologically normal (Ricucci et al. 2019). This suggests that the healthy radicular pulp could potentially be maintained when a pulpotomy procedure is performed for a vital permanent tooth clinically diagnosed with irreversible pulpitis.

Early histological studies in carious primary teeth with a history of spontaneous or lingering pain indicated extensive degenerative changes extending into the radicular pulp (Guthrie et al. 1965; Eidelman et al. 1968, 1992; Schröder 1977; Lin and Langeland 1981). The primary tooth pulp appeared to exhibit a more pronounced and widespread inflammatory reaction to a caries lesion compared to the permanent tooth pulp and was thus considered a poor candidate for VPT (Rayner and Southam 1979). However, many of the early histological studies that attempted to correlate clinical diagnosis of pulp health in primary teeth with histological findings used non-validated clinical or histological criteria (Dhillon et al. 2023).

**Fig. 1:**
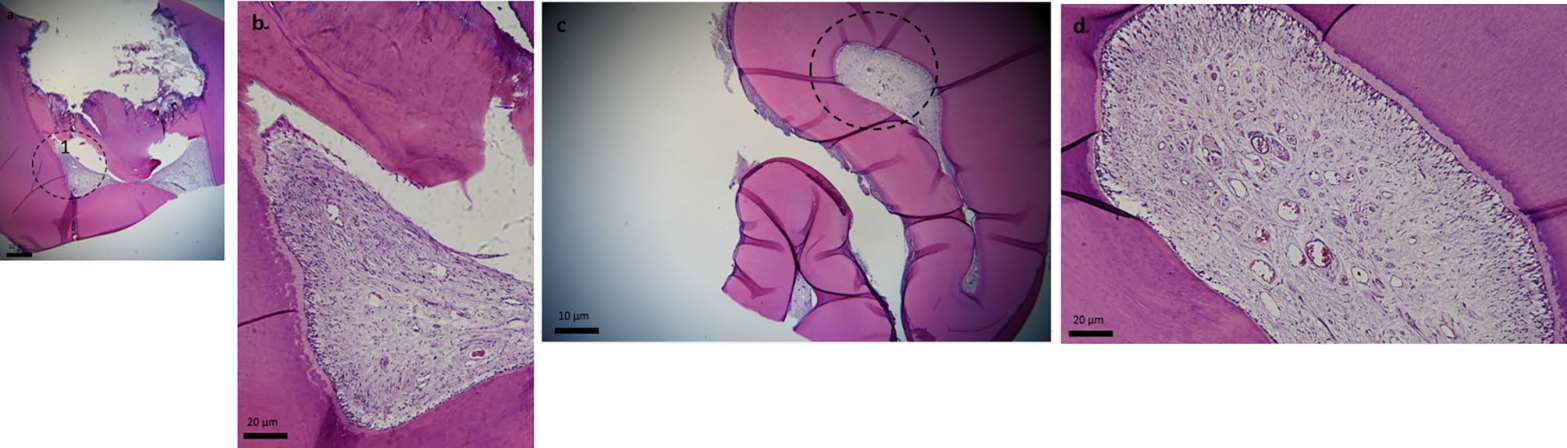
Histological sections of a tooth with clinical pulpal diagnosis of irreversible pulpitis and histological diagnosis of irreversible pulp disease in the coronal pulp and reversible pulp disease in the radicular pulp. **a** A view of the pulp chamber of an upper right first primary molar in a 5-year-old child with deep occlusal caries. **b** The mesial pulp horn is seen with tertiary dentine, areas of necrosis, and increased inflammatory cells (1) at 10X magnification. **c** Low magnification (2X) of the roots is seen here. **d** High magnification (10X) of the root. (Images and description are courtesy of Dr Ishreen Kaur Dhillon, Discipline of Orthodontics and Paediatric Dentistry, Faculty of Dentistry, National University of Singapore, Singapore.)

**Fig. 2:**
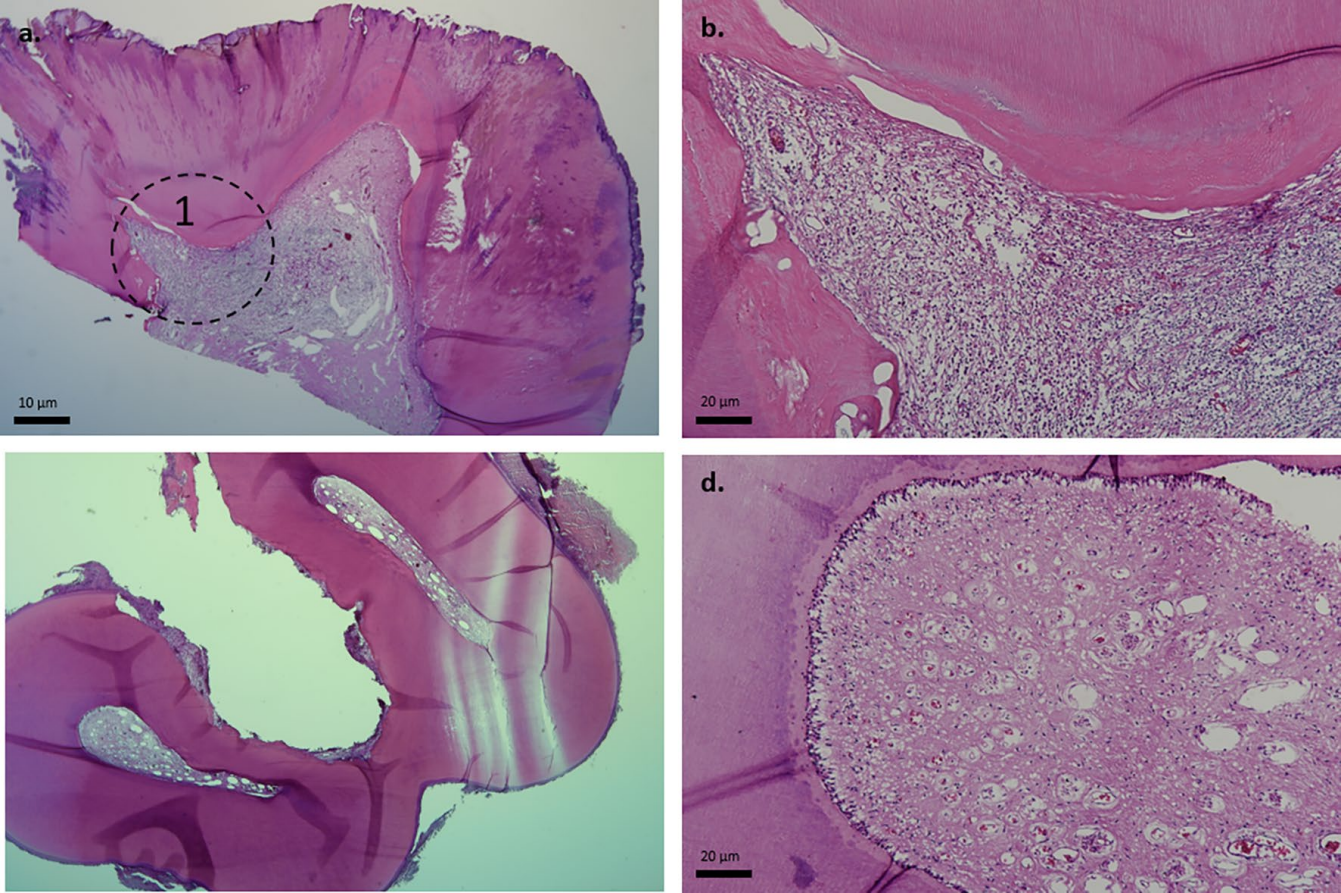
Histological sections of a tooth with clinical pulpal diagnosis of irreversible pulpitis and histological diagnosis of irreversible pulp disease in the coronal pulp. **a** A view of the pulp chamber of a lower right mandibular first primary molar in a 4-year-old child with deep multisurface caries. The mesial pulp horn with an area of necrosis (1). **b** A high magnification of the area denoted as “1” shows abundant polymorphonuclear cells. **c** Low magnification (2X) of the two roots is seen here with increased inflammatory cells. **d** and **e** High magnification (10X) of the circled region in **c** shows the mesial root with tertiary dentin (arrowhead), large vascular spaces and abundant inflammation. (Images and description are courtesy of Dr Ishreen Kaur Dhillon, Discipline of Orthodontics and Paediatric Dentistry, Faculty of Dentistry, National University of Singapore, Singapore.)

**Fig. 3:**
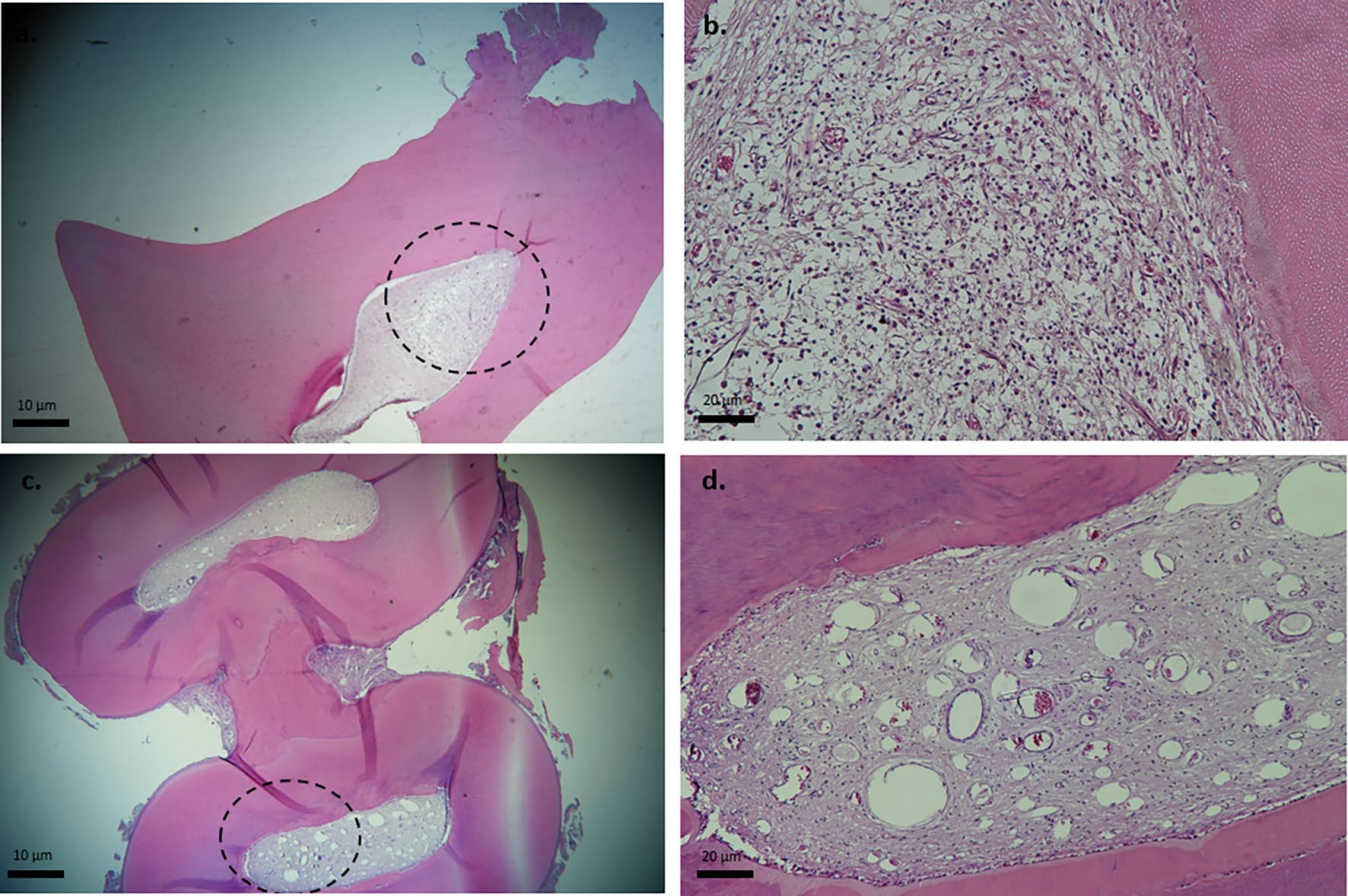
Histological sections of a tooth with clinical pulpal diagnosis of irreversible pulpitis and histological diagnosis of irreversible pulp disease in the coronal pulp. **a** A view of the pulp chamber of the lower right first primary molar in a 5-year-old child with deep multisurface caries. Circled area depicts increased inflammatory cells with no sign of viable odontoblasts. **b** High magnification (10X) of the circled area. **c** Low magnification (2X) of one of the roots is seen here with tertiary dentine and increased inflammatory cells. **d** High magnification (10X) of the circled area with large vascular spaces (Images and description are courtesy of Dr Ishreen Kaur Dhillon, Discipline of Orthodontics and Paediatric Dentistry, Faculty of Dentistry, National University of Singapore, Singapore.)

**Fig. 4:**
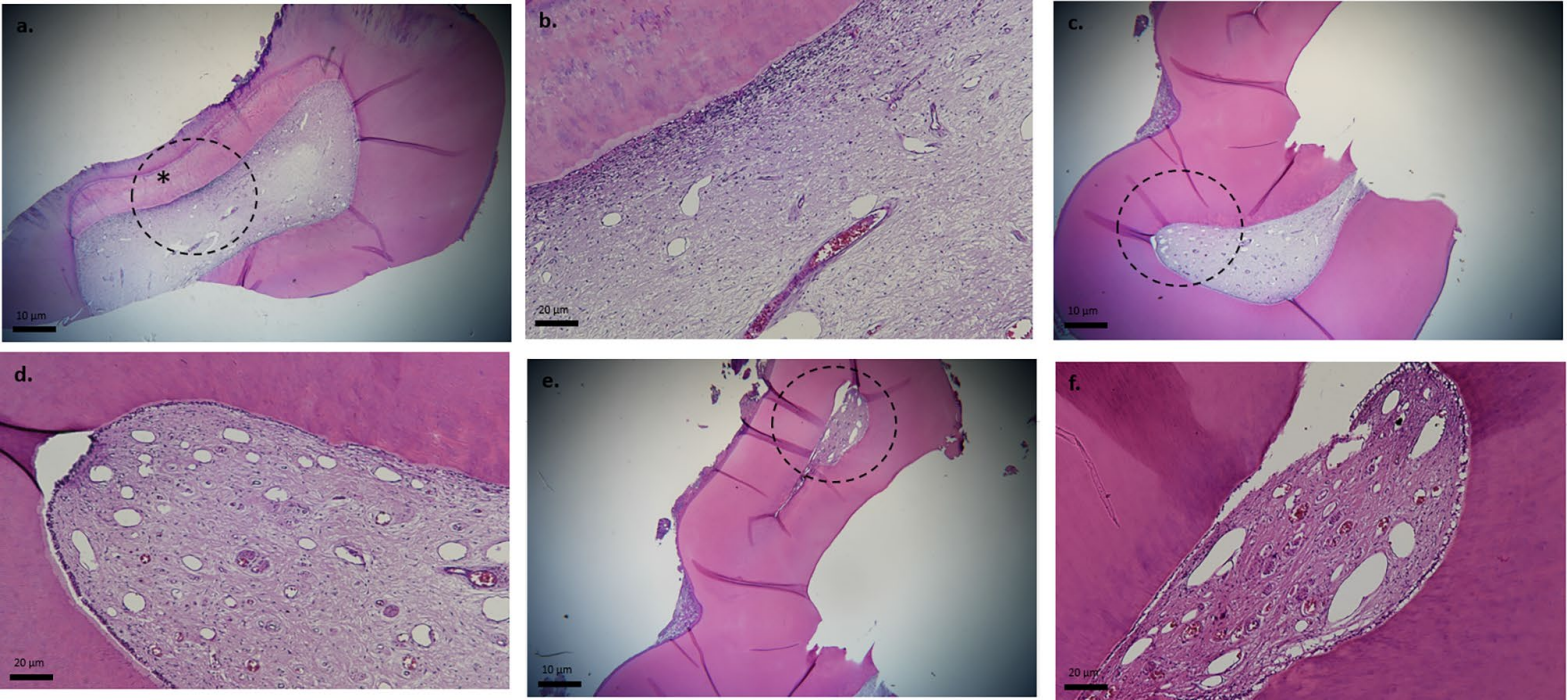
Histological sections of a tooth with clinical pulpal diagnosis of irreversible pulpitis and histological diagnosis of irreversible pulp disease in the coronal pulp and irreversible pulp disease in the radicular pulp. **a** A view of the pulp chamber of an upper left second primary molar in a 5-year-old child with deep multisurface caries. Tertiary dentine is seen (*) with increased inflammatory cells. (**b**) High magnification (10X) of the circled area. **c** Low magnification (2X) of one of the roots is seen here with normal appearance of healthy pulp cells. (**d**) **e** Low magnification (2X) of one of the roots is seen here with increased inflammatory cells. **f** High magnification (10X) of the circled area with no signs of viable odontoblast cells with numerous vascular spaces. (Images and description are courtesy of Dr Ishreen Kaur Dhillon, Discipline of Orthodontics and Paediatric Dentistry, Faculty of Dentistry, National University of Singapore, Singapore.)

Contemporary immunocytochemical and vascular studies have shown that primary and permanent tooth pulps have similar vascularity and display a comparable degree of vasodilation and angiogenesis in response to the caries insult (Rodd and Boissonade 2005, 2006). The pulpal tissues in both carious primary and permanent teeth also presented similar neural changes when mounting a defence to deep caries lesions (Rodd 2005). Moreover, although primary pulp tissue contains more immune cells in both healthy and carious states, these immune cells appear to localise in a manner similar to that seen in the permanent tooth pulp (Rodd and Boissonade 2006). A more recent histological study in primary molars demonstrated that the clinical diagnosis of irreversible pulpitis matched the histological finding of irreversible pulpal inflammation in less than half the teeth evaluated, indicating the need to refine the clinical diagnostic criteria for pulpitis-affected primary teeth (Figs. 1, 2, 3, 4) (Dhillon et al. 2023). Current understanding of the primary pulp histology and inflammatory processes does indicate the potential for pulp healing and repair even if the primary tooth pulp is clinically diagnosed to be ‘irreversibly’ inflamed.

## Diagnostic considerations

The AAE diagnostic terminology assigns vital pulp to one of three categories: “normal”, “reversible pulpitis” or “irreversible pulpitis” (Levin et al. 2009). The designation of a specific pulpal diagnosis is based on the patient’s pain history and appropriate clinical testing. Systematic reviews suggest that the diagnostic accuracy of clinical symptoms/signs or sensibility tests to distinguish between reversible or irreversible pulp inflammation was unsatisfactory (Mejàre et al. 2012; Mainkar and Kim 2018). Thus, clinical diagnosis of pulpal diseases is only an ‘educated’ judgement as the correlation between clinical symptoms/signs and the actual histological picture is not straightforward. Based on this evolving evidence, the ESE position paper states that ‘reversible and irreversible pulpitis diagnoses are clinical terms that are operational but not biological’ (Duncan et al. 2019). Consequently, new diagnostic terminologies have been proposed for pulpitis-affected permanent teeth to give a more accurate clinical reflection of the histological picture (Wolters et al. 2017). It has also been suggested that the diagnostic term for pulpal inflammation should be confined to only ‘pulpitis’ without further designation until intra-operative confirmation (Taha et al. 2020).

For primary teeth, clinically identifying reversible or irreversible pulpitis depends almost entirely on the patient’s subjective symptoms as pulp sensibility testing or percussion tests are not very reliable in children. However, whilst older children are likely able to give a reasonable description of their pain history, eliciting an accurate one in young children is a challenge and often dependent on information provided by the caregiver, potentially compromising the value of pain experience in making an accurate clinical diagnosis of pulpitis-affected primary teeth. The traditional diagnostic criteria to distinguish between reversible pulpitis, irreversible pulpitis and pulpal necrosis in primary teeth are outlined in Table 1. Clinicians need to be aware of the potential to misdiagnose primary tooth pulpitis when using the traditional criteria. Considering the improved understanding of primary pulp biology, the terms “reversible pulpitis” and “irreversible pulpitis” are putatively obsolete as they do not depict an accurate clinical reflection of the histological situation. A new diagnostic nomenclature for inflamed primary tooth pulp based on clinical symptoms/signs, their possible histological characteristics, and the treatment options suggested for these conditions is presented in Table 2. This proposed diagnostic nomenclature for primary tooth pulpitis is adapted from new diagnostic terminologies suggested for permanent tooth pulpitis (Wolters et al. 2017). However, the proposed diagnostic system for primary tooth pulpitis requires validation and prospective testing before widespread implementation.

**Table 1:** Diagnostic criteria for pulpitis and necrosis in primary teeth

	Reversible pulpitis	Irreversible pulpitis	Pulp necrosis
Lingering pain to thermal stimuli	Absent	Present	Absent
Spontaneous pain	Absent	Likely	Possible
Sleep disturbance	Absent	Possible	Possible
Percussion tenderness	Unlikely	Possible	Possible
Palpation tenderness	Absent	Possible	Possible
Associated swelling/sinus tract	Absent	Absent	Likely
Periapical radiolucency	Absent	Absent	Possible
Furcal radiolucency	Unlikely	Possible	Present

**Table 2:** Proposed diagnostic nomenclature for assessing pulpitis in primary teeth and subsequent treatment needs

Pulp status	Clinical/radiographic picture	Possible histological picture	Treatment options suggested
Initial pulpitis	Heightened but non-lingering response to thermal stimuli No spontaneous pain or percussion sensitivity Carious lesion extending to the inner third of the dentine	Limited local inflammation confined to coronal pulp	Indirect pulp capping and composite restoration ensuring leakproof coronal seal Restore with a PMC using the conventional or Hall technique
Mild pulpitis	Heightened lingering response to thermal stimuli lasting up to 20 s No spontaneous pain, but possible percussion sensitivity Carious lesion extending to the inner third of the dentine	Limited local inflammation confined to coronal pulp	Indirect pulp capping and composite restoration ensuring leak-proof coronal seal Restore with a PMC using the conventional or Hall technique
Moderate pulpitis	Strong, heightened and lingering response to thermal stimuli which can last for minutes Spontaneous dull pain that is controlled with analgesics Possibly percussion sensitive Carious lesion extending to pulp	Extensive local inflammation confined to coronal pulp	Full coronal pulpotomy and conventional PMC restoration
Severe pulpitis	Clear pain reaction to thermal stimuli Severe spontaneous sharp or dull pain with limited relief from analgesics Very sensitive to percussion Carious lesion extending to pulp	Extensive local inflammation of coronal pulp possibly extending into radicular pulp	Full coronal pulpotomy if haemostasis can be achieved within 10 min If bleeding from pulp stumps persists beyond 10 min full pulpectomy or extraction is indicated

Adapted from Wolters et al. 2017. *PMC* pre-formed metal crown

## State of evidence

Pulpotomy has traditionally not been part of the treatment considerations for primary teeth diagnosed with irreversible pulpitis; however, there is now increasing evidence to suggest that pulpotomy is a successful treatment option to manage vital primary molars diagnosed with irreversible pulpitis. To summarise and contextualise the current state of evidence, a structured and transparent literature search was undertaken to identify relevant studies and minimise selection bias. This approach was designed to strengthen the methodological clarity of this narrative review, rather than to constitute a formal systematic review. The search was conducted up to January 2026 and seven studies were accordingly identified (Supplemental Fig. 1). The primary search was conducted on four electronic databases (MEDLINE, Embase, Web of Science, and Cochrane Library). The search terms used included: ‘pulpotomy’, ‘coronal pulpotomy’, ‘pulpectomy’, ‘extraction’, ‘irreversible pulpitis’, ‘primary teeth’, ‘primary molars’, ‘deciduous teeth’, and ‘deciduous molars’. The search terms were merged using Boolean operators ‘AND’ and ‘OR’ to form tailored database queries. Besides the electronic database searches, references of the included studies were also manually screened to find any missing eligible publications. The research question was tailored through the following PICO framework: population (P): children with primary teeth clinically diagnosed with irreversible pulpitis; intervention (I): pulpotomy; comparisons (C): none, pulpectomy, or tooth extraction; and outcome (O): clinical and radiographic success. The inclusion criteria for study selection were: (1) pulpotomies in primary teeth with clinical signs and symptoms of irreversible pulpitis; (2) randomised controlled trials, non-randomised clinical trials, prospective/retrospective cohort studies, and case–control studies; (3) studies reporting clinical/radiographic outcomes with a minimum 6-month post-treatment follow-up; and (4) studies published in English language. Extracted data included study characteristics like country/year, study design, sample size, patient age, type of tooth treated, and treatment-related details (haemostasis agent/time, pulpotomy medicament, final restoration type, follow-up periods, and clinical/radiographic outcomes).

The methodological details of seven studies that investigated pulpotomy treatment for vital primary molars diagnosed with irreversible pulpitis are presented in Table 3. Sodium hypochlorite (NaOCl) or saline were the haemostatic agents used in these studies and the haemostasis time ranged from 2 to 10 min. The majority of the studies used different types of calcium silicate cements (CSCs) as the pulpotomy medicament over the radicular pulp stumps and preformed metal crowns (PMCs) were the preferred post-pulpotomy final restoration. The clinical and radiographic treatment outcomes of the seven studies where pulpotomy was used for the management of primary molars clinically diagnosed with irreversible pulpitis are detailed in Table 4. Studies where CSCs were used as the pulpotomy medicament showed clinical success rates ranging from 95 to 100%, 1 year post-treatment. The radiographic success at this time point ranged from 90 to 100%.

**Table 3:** Studies investigating pulpotomy for management of vital primary molars with irreversible pulpitis

Study title	Country year	Study design patient age	Teeth treated sample size of pulpotomy arm	Haemostasis agent and time	Pulpotomy medicament and final restoration	Comparators
Pulpotomy for treating vital primary molars with clinical symptoms of irreversible pulpitis—a prospective cohort study (Philip et al. 2025)	5 countries 2025	PC (NRCT) 4 to 9 years	Primary molars 50	2% NaOCl Up to 6 min	MTA PMC	None
Comparative evaluation of cervical pulpotomy and pulpectomy for primary molars with irreversible pulpitis: a multicentre randomised controlled trial (Sabbagh et al. 2024)	Iran 2024	RCT 4 to 9 years	Primary molars 74	Saline Up to 10 min	CEM PMC	CEM pulpotomy vs. Metapex pulpectomy
Evaluation of the efficacy of mineral trioxide aggregate and bioceramic putty in primary molar pulpotomy with symptoms of irreversible pulpitis: a randomised-controlled trial (Al-Nassar et al. 2023)	Syria 2023	RCT 6 to 8 years	Primary lower molars 40	2.5% NaOCl 2 min	MTA and BCP PMC	MTA pulpotomy vs. BCP pulpotomy
A retrospective study of iRoot BP Plus pulpotomy compared with Vitapex pulpectomy for irreversible pulpitis of primary molars with the presence of coronal pulp tissue (Hu et al. 2023)	China 2023	RC (NRCT) 3 to 7 years	Primary molars 88	3% NaOCl 5–10-min	iRoot BP plus CR or PMC	iRoot Pulpotomy vs. Vitapex pulpectomy
Comparison of clinical and radiographic success between MTA and Biodentine in pulpotomy of primary mandibular second molars with irreversible pulpitis: a randomised double-blind clinical trial (Eshghi et al. 2022)	Iran 2022	RCT 3 to 6 years	Primary lower second molars 52	Saline 5 min	MTA and BD PMC	MTA pulpotomy vs. BD pulpotomy
A comparative clinical radiological study between using formocresol, MTA and platelet-rich fibrin (PRF) in pulpotomy of second primary molars (Al-Awwad et al. 2021)	Syria 2021	PC (NRCT) 5 to 9 years	Primary upper and lower second molars 36	Saline 5–10 min	MTA, FC and PRF PMC	FC pulpotomy vs. MTA pulpotomy vs. PRF pulpotomy
Calcium-enriched mixture pulpotomy of primary molar teeth with irreversible pulpitis: a clinical study (Memarpour et al. 2016)	Iran 2016	PC (NRCT) 6 to 8 years	Primary molars 50	Saline 5 min	CEM PMC	None

*PC* prospective cohort, *NRCT* non-randomised clinical trial, *RCT* randomised clinical trial, *RC* retrospective cohort, *MTA* mineral trioxide aggregate, *CEM* calcium-enriched mixture, *BCP* bioceramic putty, *BD* biodentine, *FC* formocresol, *PRF* platelet-rich fibrin

**Table 4:** Clinica/radiographic treatment outcomes of pulpotomy for management of vital primary molars with irreversible pulpitis

Study title	Pulpotomy medicament	Clinical success of pulpotomy		Radiographic success of pulpotomy	
		6-m	≥ 12-m	6-m	≥ 12-m
Pulpotomy for treating vital primary molars with clinical symptoms of irreversible pulpitis—a prospective cohort study (Philip et al. 2025)	MTA	100%	–	93.9%	–
Comparative evaluation of cervical pulpotomy and pulpectomy for primary molars with irreversible pulpitis: a multicentre randomised controlled trial (Sabbagh et al. 2024)	CEM	98.6%	97.1%	98.6%	96.4%
Evaluation of the efficacy of mineral trioxide aggregate and bioceramic putty in primary molar pulpotomy with symptoms of irreversible pulpitis: a randomised-controlled trial (Al-Nassar et al. 2023)	MTA and BCP	MTA 95% BCP 100%	MTA 95% BCP 100%	MTA 95% BCP 100%	MTA 95% BCP 100%
A retrospective study of iRoot BP Plus pulpotomy compared with Vitapex pulpectomy for irreversible pulpitis of primary molars with the presence of coronal pulp tissue (Hu et al. 2023)	iRoot BP plus	–	98.9% at 18-m	–	95.5% at 18-m
Comparison of clinical and radiographic success between MTA and Biodentine in pulpotomy of primary mandibular second molars with irreversible pulpitis: a randomised double-blind clinical trial (Eshghi et al. 2022)	MTA and BD	MTA 96.15% BD 100%	MTA 96.15% BD 96.15%	MTA 100% BD 96.15%	MTA 96.15% BD 96.15%
A comparative clinical radiological study between using formocresol, MTA and platelet-rich fibrin (PRF) in pulpotomy of second primary molars (Al-Awwad et al. 2021)	MTA, FC and PRF	MTA 100%, FC 75%, PRF 91.7%	–	–	–
Calcium-enriched mixture pulpotomy of primary molar teeth with irreversible pulpitis: a clinical study (Memarpour et al. 2016)	CEM	97.8%	92.8%	97.8%	90.4%

*MTA* mineral trioxide aggregate, *CEM* calcium-enriched mixture, *BCP* bioceramic putty, *BD* biodentine, *FC* formocresol, *PRF* platelet-rich fibrin

A systematic review and meta-analysis of five of these studies, involving 266 primary teeth amongst children aged 3–9 years, concluded that pulpotomy could be a prospective alternative to conventional pulpectomy for managing primary teeth diagnosed with irreversible pulpitis (Lin et al. 2024). An even more recent systematic review and meta-analysis compared the clinical and radiographic success outcomes of pulpotomy versus pulpectomy in primary teeth with irreversible pulpitis and concluded that pulpotomy was an effective, minimally invasive alternative to pulpectomy (Chawla et al. 2026)

## Case selection

Clinical studies have shown that painful pulpitis is not necessarily a contraindication to VPT in primary and permanent teeth (Taha et al. 2020; Sabbagh et al. 2024; Philip et al. 2025). However, careful case selection is required when considering pulpotomy as a treatment option to manage teeth clinically diagnosed with irreversible pulpitis. The pre-operative and intra-operative signs and symptoms that can serve as a guideline in the choice of pulpotomy over pulpectomy/extraction for irreversible pulpitis-affected primary molars include:

- Clinical symptoms typical of irreversible pulpitis such as severe lingering or spontaneous pain.
- No clinical signs of pulp infection such as pathologic tooth mobility, soft tissue swelling or associated sinus tract.
- Pre-operative radiograph shows advanced carious lesion approaching the pulp with no signs of periapical radiolucency or pathological root resorption.
- Intra-operatively, healthy vital pulp is seen after pulp chamber deroofing, presenting as uniformly reddish pink vascular tissue.
- Intra-operatively, after coronal pulp amputation, radicular pulp haemostasis is achieved within 10 min (depending on child’s cooperation). The time taken to achieve haemostasis has long been considered a surrogate indicator for the degree of pulpal inflammation and as a prognostic factor for procedural success of VPT (Matsuo et al. 1996). However, the reliability of using haemostasis time as an indicator for pulpal inflammatory status has been questioned for both primary and permanent teeth (Mutluay et al. 2018; Zanini et al. 2019). Successful pulpotomy outcomes for managing irreversible pulpitis in permanent teeth have been reported for bleeding times ranging from 1 to 25 min (Linsuwanont et al. 2017; Taha et al. 2020), and up to 10 min for primary teeth (Sabbagh et al. 2024; Philip et al. 2025). Based on these study results, the established practice of ruling out pulpotomy in primary teeth if radicular pulp haemostasis is not achieved within 5 min may need reconsideration. However, extending haemostasis time beyond 10 min during a pulpotomy procedure for ambulant children may not be practically feasible.

## Proposed procedural protocol

Whilst the pulpotomy procedure is less challenging than conventional pulpectomy, it still requires strict adherence to procedural guidelines for long-term success as detailed below:

### Pre-operative clinical assessment

Clinical symptoms/signs of irreversible pulpitis may include the following:

(i) Strong heightened lingering response to thermal stimuli that can last for minutes.
(ii) Spontaneous pain which can be worse at night.
(iii) Referred pain which may be difficult to localise.

### Pre-operative radiographic assessment

Periapical radiography of the affected primary tooth is essential for assessing the following:

(iv) Carious lesion depth: Teeth with irreversible pulpitis symptoms will show advanced carious lesions possibly extending into the pulp on two-dimensional radiographs.
(v) Furcal radiolucency: The near equivalent of periapical radiolucency observed in permanent teeth is the furcal radiolucency often seen in cariously exposed primary molars. Periapical healing following pulpotomy in permanent teeth with periapical radiolucencies has been reported in several studies (Linsuwanont et al. 2017; Qudeimat et al. 2017; Taha and Abdelkhader 2018). Similarly, for primary teeth, early-stage furcal radiolucency likely indicates release of inflammatory products through the porous pulp chamber floor and not necessarily necrotic pulp. In such cases, preliminary studies have shown that pulpotomy is likely to be a relevant treatment option as long as the pulp is assessed intra-operatively to be vital and haemostasis is achieved (Philip et al. 2025). However, extensive furcal radiolucency is likely to indicate non-vital pulp or extensive pulpal inflammation, ruling out VPT as a treatment option.
(vi) Root resorption status: Any signs of pathological root resorption indicate that the primary tooth is not suitable for VPT or even pulpectomy. Additionally, pulpotomy in primary teeth is generally indicated only if any physiological root resorption is limited to less than one-third of the root length.
(vii) Periapical health status: Periapical lesions in relation to the primary tooth roots most likely indicate pulp non-vitality and pulpotomy is contra-indicated.
(viii) Eruption status of the permanent successor: pulp therapy is normally not required if the affected primary tooth is likely to be replaced by its permanent successor within 6 months and the painful tooth can be extracted.

### Aseptic operative technique

Successful outcomes for pulpotomy are contingent on strict adherence to aseptic operative techniques including:

(i) Mandatory rubber dam isolation: The rubber dam facilitates a clean operative environment that is critical for VPT success besides protecting the patient from procedural dangers.
(ii) Carious tissue excavation: It is important to remove as much carious tissue as possible to minimise further bacterial contamination of the pulp. Carious tissue excavation should start at the cavity periphery and then progressively over the pulp chamber roof.

### Pulp chamber deroofing and vitality assessment

(i) After initial entry into the pulp chamber, a fresh sterile safe-ended bur can be used to deroof the chamber by carefully removing the ceiling of dentine, ensuring no interferences or overhangs remain over the pulp chamber.
(ii) Following deroofing, pulp vitality should be confirmed visually, preferably under magnification. Healthy vital pulp will appear as uniformly reddish pink vascular tissue and the tooth is considered appropriate for pulpotomy. Non-vital necrotic pulp will present as pale or very dark avascular tissue with minimal bleeding or as yellowish or greyish liquefied areas and pulpectomy or extraction is indicated in such cases.

### Pulp amputation and haemostasis

(ii) Removal of the inflamed and infected coronal pulp tissue can be done using a sharp spoon excavator or a slow-speed rotatory bur. The endpoint of this coronal pulp amputation should be at the root canal orifices.
(iii) Light pressure compression using a 0.5 to 5% NaOCl-soaked cotton pellet is recommended for disinfection, removing adherent biofilms, and achieving haemostasis at the amputation site. The use of lower concentrations is usually recommended in children, especially in less cooperative children. NaOCl is preferred over physiological saline, which lacks disinfection properties (Tüzüner et al. 2012), or haemostatic agents like ferric sulphate, which can mask the true pulp inflammatory status (Duncan et al. 2019). The concentration of NaOCl to be used does not appear to be a prognostically decisive factor (Munir et al. 2020).
(iv) Radicular pulp haemostasis should be assessed and achieved within 10 min (depending on child’s cooperation).

### Pulpotomy medicament and coronal restoration

Probably the most critical factor in achieving favourable pulpotomy outcomes is adequate sealing of the remnant pulp tissue with a bioactive hydrophilic medicament and a definitive coronal restoration.

(i) Once radicular pulp haemostasis is achieved, 2–3 mm of a hydrophilic and dimensionally stable capping material should be directly adapted over the pulp stumps, ensuring there is no porosity or any excess cement on the pulp chamber walls. Clinical trials comparing different pulpotomy medicaments have shown that, irrespective of the medicament used, the pulpotomy procedure was clinically and radiographically successful in treating primary molars diagnosed with irreversible pulpitis (Al-Awwad et al. 2021; Eshghi et al. 2022; Al-Nassar et al. 2023). New-generation bioactive calcium silicate cements are not only dimensionally stable with excellent sealing abilities, but also have biocompatible, immunomodulatory and osteogenic properties (Parirokh et al. 2018). These materials have been shown to induce the release of regenerative dentine-bound growth factors, upregulate angiogenesis, and stimulate cellular differentiation of dentine-forming cells (Smith et al. 2016; Tomson et al. 2017).
(ii) Immediate placement of a definitive coronal restoration is recommended to prevent microleakage, protect the bioactive medicament, and reduce any post-operative sensitivity. For children, PMCs are an excellent full coverage restorative option having demonstrated high success rates for pulpotomised primary molars and first permanent molars that were diagnosed with irreversible pulpitis (Qudeimat et al. 2017; Sabbagh et al. 2024; Philip et al. 2025). Preformed zirconia crowns could be an aesthetic alternative to PMCs offering superior seal and preservation of coronal integrity compared to intra-coronal restorations (Foster et al. 2024).

### Follow-up assessment

The ESE position paper recommends that permanent teeth that receive VPT should be assessed with clinical, radiographic and sensibility testing at 6 and 12 months post-operatively, and thereafter at yearly intervals for up to 4 years (Duncan et al. 2019). A similar follow-up protocol can be followed for primary teeth with assessment of treatment success based on clinical and radiographic outcome criteria detailed below:

(i) The clinical outcome measures for success are an asymptomatic functional tooth with no tenderness to percussion or palpation and no swelling or sinus tract associated with the treated tooth.
(ii) Radiographically, there should be no signs of pathologic root resorption or periapical lesions and evident healing or no worsening of any pre-operative furcal lesions.

## Conclusions

Ideally, primary teeth with symptoms of irreversible pulpitis need to be treated in a way that is most likely to keep them free of symptoms until their natural exfoliation. Pulpotomy is increasingly considered as a therapeutic alternative to traditional pulpectomy to treat primary teeth diagnosed with irreversible pulpitis. This evolving change is based on the drive to practise minimally invasive endodontics, improved understanding of pulp biology, and accumulating evidence from clinical and radiographic outcome studies. However, more high-quality comparative research is warranted to determine the long-term success of pulpotomy, refine treatment protocols, and develop objective diagnostic measures of the pulpal conditions affecting primary teeth.

The authors therefore call for an update of international guidelines to incorporate a revised diagnostic framework that recognises the different stages of pulpitis in primary teeth, alongside the adoption of minimally invasive treatment approaches. Such an update should take into account the emerging evidence supporting the success of pulpotomy in the management of primary teeth clinically diagnosed with irreversible pulpitis, thereby reducing unnecessary invasive interventions, including pulpectomy and extraction.

## Supplementary Information

Below is the link to the electronic supplementary material.

Supplementary file1 (PNG 38 KB)

## Data Availability

No datasets were generated or analysed during the current study.
